# Coronary Events in Patients Presenting for Repair of Acute Type A Aortic Dissection

**DOI:** 10.12945/j.aorta.2017.16.058

**Published:** 2018-09-24

**Authors:** Paul C. Tang, Shahab A. Akhter, Satoru Osaki, Lucian Lozonschi, Takushi Kohmoto, Nilto C. De Oliveira

**Affiliations:** 1 Department of Surgery, Division of Cardiothoracic Surgery, University of Wisconsin School of Medicine and Public Health, Madison, Wisconsin, USA

**Keywords:** Aorta, Thoracic, Aortic dissection, Coronary artery disease, Coronary artery imaging, Coronary syndromes, Myocardial ischemia

## Abstract

***Background:*** Preoperative coronary angiography is often not performed in acute Type A dissection. We examined differences in the incidence of pre-existing coronary disease and subsequent coronary events between patients undergoing acute Type A dissection repair and patients undergoing elective proximal aortic aneurysm repair.

***Methods:*** From 2000 to 2015, there were 154 acute Type A dissection repairs and 457 elective proximal aortic aneurysm repairs. We performed a retrospective review to evaluate preoperative coronary disease and postoperative coronary interventions such as percutaneous coronary intervention (PCI) and coronary bypass grafting (CABG).

***Results:*** A total of 31 (20%) dissection patients and 123 (27%) elective surgery patients had preoperative evidence of coronary artery disease (p = 0.094). All elective surgery patients but only six (4%) dissection patients had preoperative coronary catheterization. More CABGs were performed in the elective surgery group (19%) than in the dissection group (3%, p < 0.001). There were no differences in the incidence of prior PCI, CABG, or myocardial infarction between groups. Following dissection repair, four patients required coronary interventions. Of these, two (1.3%) experienced chest pain and underwent PCI at 4.7 and 4.3 months postoperatively, respectively, and another two experienced symptoms and required PCI at 5 and 7 years, respectively. The 30-day and 14-year mortality rates after dissection repair were 13% and 24%, respectively. Although the dissection group had poorer survival than the elective surgery group (p < 0.001), there was no difference in conditional survival after aortic-related deaths over the first year were censored (p = 0.104).

***Conclusions:*** Given the low incidence of missed significant coronary disease (1.3%), it is reasonable to perform Type A dissection repair without coronary angiography.

## Introduction

Acute Type A dissection is an emergent condition that requires timely operative intervention. While aortography allows the diagnosis of concomitant native coronary artery disease during evaluation for dissection, the diagnosis of acute aortic dissection has largely been supplanted by computed tomographic angiography and sometimes magnetic resonance imaging 1 , largely due to the widespread accessibility and reproducibility of these modalities. Although these techniques may identify proximal coronary artery involvement, they are unable to evaluate more distal disease. However, transesophageal echocardiography during the course of dissection evaluation can identify regional wall motion abnormalities in addition to ostial coronary obstruction that may indicate coronary compromise.

The incidence of acute myocardial ischemia due to coronary malperfusion is around 5–6% 2 3 4 . In an autopsy series, Larson and Edwards found critical (i.e., grade 4) coronary artery disease in 27 out of 121(22%) patients who had Type I or II aortic dissection 5 . Rizzo et al. found high operative mortality among patients with Type A dissection who had preoperative angiography, whereas there were no deaths due to aortic rupture or coronary artery disease complications among patients taken directly to surgery following noninvasive diagnosis of acute Type A dissection 6 . By contrast, Penn et al. found that angiographic delineation of coronary anatomy did not negatively impact operative survival, although this study did not include patients who died while waiting for angiography and subsequent surgery 7 .

At our institution, we prefer the expeditious direct transfer of patients to the operating room. Following repair of Type A dissection, coronary evaluation is not usually routinely performed unless the patient is symptomatic. In the present study, we evaluated the short- and long-term outcomes of this approach and subsequent postoperative coronary interventions. Our control group consisted of patients who underwent elective ascending aortic aneurysm repair in which preoperative coronary catheterization was mandatory. The validity of this group as a control is based on the assumption that a significant portion of Type A dissections result from pre-existing ascending aortic aneurysms.

## Materials and Methods

### Patients

This study was approved by the University of Wisconsin-Madison Institutional Review Board and conforms to the provisions of the Declaration of Helsinki. We analyzed the records of 154 patients who underwent consecutive acute Type A aortic dissection repair and 457 patients who underwent elective proximal aortic aneurysm repair at University of Wisconsin hospitals and clinics between January 2000 and July 2015. Stanford Type A dissection was defined as acute if the onset of symptoms was less than 14 days from the time of surgery. A retrospective review was performed for preoperative coronary disease based on prior coronary catheterization, stress tests, and history of coronary events such as myocardial infarction (MI). We also analyzed patient age, gender, comorbidities, creatinine, left ventricular ejection fraction (LVEF), operative variables, postoperative complications, and survival. Postoperative coronary studies and interventions such as stress tests, coronary catheterization, percutaneous coronary interventions (PCI), and coronary artery bypass grafting (CABG) were examined. All elective surgery patients and 6 out of 154 (4%) dissection patients received preoperative coronary evaluation (p < 0.001). For patients in the dissection group who did not undergo preoperative coronary angiography, coronary evaluation was performed intraoperatively by visually inspecting the coronary ostia after opening the aorta. For patients in the dissection group who did undergo preoperative coronary angiography, two patients had no coronary disease; one patient had prior CABG, with catheterization demonstrating all patent grafts; one patient had right coronary malperfusion from the dissection flap; and two patients had significant underlying atherosclerotic coronary disease.

### Follow-up

Survival data were available for all patients and were obtained through detailed clinical follow-up. Follow-up is expressed in years using mean and standard deviation. Maximum follow-up was 14.04 years, with a total follow-up of 1948.48 patient years. Aortic-related deaths are defined as those that occurred as a complication of the initial surgery for aortic-related pathology or from any residual aortic disease (e.g., residual Type B dissection).

### Statistical Analysis

Pearson Chi-square or Fisher’s exact tests were used to analyze categorical variables. Kaplan-Meier survival curves with Mantel-Cox statistics were used to analyze survival data. Student’s t-tests were used to analyze continuous variables. Statistical analysis was performed using SPSS software (SPSS Inc., Chicago, IL).

## Results

### Patient Demographics and Coronary Findings

There were no differences in patient age, gender, LVEF, or other comorbidities between the dissection and elective surgery groups (p > 0.05, Table 1 ). The lack of difference in a history of cancer within 5 years of surgery suggests that any differences in survival were unlikely due to cancer. However, creatinine was higher in the dissection group (1.2 ± 0.6) than in the elective surgery group (1.1 ± 0.5, p = 0.018). Coronary evaluation ( Table 2 ) demonstrated no difference in history of previous PCI, MI, or CABG (p > 0.05). There was no difference in the number of patients with known preoperative coronary artery disease between the dissection (31 out of 154, 20%) and elective surgery (123 out of 457, 27%) groups (p = 0.094). In the elective surgery group, 85 out of 457 (19%) patients required CABG for atherosclerotic coronary disease. Of these, 57 out of 457 (12%) required a graft to the left anterior descending artery. Most patients (44 out of 457, 10%) in the elective surgery group needed one coronary graft, whereas 26 (6%) needed two grafts. In the dissection group, four concomitant CABGs were performed. Of these four patients, one was found to have a right coronary artery ostial stenosis noted visually upon opening the ascending aorta; one patient had preoperatively identified left anterior descending artery disease on coronary angiography; one received three-vessel CABG for dissection of the left main and right coronary artery; and one received empiric bypass grafts to the left anterior descending and obtuse marginal arteries due to unexplained poor anterolateral wall function on cardiac reperfusion. This last patient had no direct visual evidence of coronary dissection or technical issues with the coronary button, so poor cardiac function after cross clamp removal may have resulted from undiagnosed underlying atherosclerotic coronary artery disease. This patient’s cardiac function improved after CABG, and the patient ultimately survived.

**Table 1.** Patient demographics.

Variable	Type A Dissection Repair (n = 154)	Elective Proximal Aortic Surgery (n = 457)	*P* -value
Age (years)	61.4 ± 14.3	59.8 ± 14.1	0.229
Sex (male)	106 (69%)	335 (73%)	0.284
Body mass index	29.2 ± 6.5	29.1 ± 6.0	0.867
Preoperative creatinine (mg/dL)	1.2 ± 0.6	1.1 ± 0.5	0.018
LVEF (%)	58.2 ± 9.3	59.0 ± 10.3	0.403
Hypertension	110 (71%)	305 (67%)	0.281
Dialysis	2 (1%)	4 (1%)	0.645
Cerebrovascular disease	13 (8%)	32 (7%)	0.554
Peripheral vascular disease	38 (25%)	81 (18%)	0.060
Lung disease	29 (19%)	102 (22%)	0.362
Liver disease	2 (1%)	2 (0.4%)	0.252
Diabetes	12 (8%)	35 (8%)	0.957
Hyperlipidemia	70 (45%)	224 (49%)	0.444
Cancer within 5 years of surgery	3 (2%)	8 (2%)	0.873

Nominal data are presented as frequency (n) and percentage of the total population and were analyzed using Pearson Chi-square or Fisher’s exact tests. Continuous data are presented as mean ± standard deviation and were analyzed using two-tailed paired Student’s t-tests. LVEF= left ventricular ejection fraction.

**Table 2.** Coronary artery disease history, evaluations, and interventions.

Variable	Type A Dissection Repair (n = 154)	Elective Proximal Aortic Surgery (n = 457)	*P* -value
Previous PCI	8 (5%)	23 (5%)	0.937
Previous CABG	5 (3%)	6 (1%)	0.119
Prior MI	15 (10%)	43 (9%)	0.904
Number of preoperatively known diseased coronary vessels			0.038
0	134 (87%)	355 (77%)	
1	11 (7%)	49 (11%)	
2	2 (1%)	28 (6%)	
3	7 (5%)	25 (5%)	
Concomitant CABG	4 (3%)	85 (19%)	< 0.001
Total grafts			0.001
0	150 (97%)	372 (81%)	
1	2 (1%)	44 (10%)	
2	1 (0.6%)	26 (6%)	
3	1 (0.6%)	11 (2%)	
4	0 (0%)	1 (0.2%)	
5	0 (0%)	1 (0.2%)	
6	0 (0%)	2 (0.4%)	
Graft to LAD	3 (2%)	57 (12%)	< 0.001

Nominal data are presented as frequency (n) and percentage of the total population and were analyzed using Pearson Chi-square or Fisher’s exact tests. CABG = coronary artery bypass graft; LAD = left anterior descending artery; MI = myocardial infarction; PCI = percutaneous coronary intervention.

Following Type A dissection repair, four patients required subsequent coronary interventions. Of these, two (1.3%) experienced chest pain following discharge and underwent coronary catheterization with stent placement at 4.7 and 4.3 months after the operation, respectively. Stents were placed in the left anterior descending artery in the first patient and in the left anterior descending and left main arteries in the second patient. Another two patients experienced symptoms consistent with progression of native atherosclerotic coronary disease and required coronary stent placement at 5 and 7 years after dissection repair, respectively. We identified five coronary malperfusions (3.2%) upon presentation. All four patients with malperfusion and confirmed involvement of the left coronary artery died during their hospital stay, and one patient with malperfusion involving the right coronary artery was alive at 5 years. No patient presented with new MI after 30 days postoperatively.

### Operative Parameters

There was no difference in median operative year, which was 2010 ( Table 3 ), suggesting that operative techniques as well as pre- and postoperative management were likely comparable between groups. There were more reoperative procedures in the elective surgery group (15%) than in the dissection group (6%, p = 0.007). No group difference in concomitant mitral or tricuspid valve surgery was found (p > 0.05). There were more hemi- and total arch replacements in the dissection group (p < 0.001) and more ascending aortic replacements without arch repair in the elective surgery group (p < 0.001). There were more composite valve graft root and aortic valve replacements in the elective surgery group (p < 0.001) and more aortic valve repairs (mostly aortic valve resuspensions) in the dissection group (p < 0.001). The cross-clamp time was higher in the elective surgery group (130 ± 50 min) than in the dissection group (104 ± 47 min, p < 0.001), probably due to the higher number of composite valve graft root and aortic valve replacements in the elective surgery group. However, cardiopulmonary bypass time was higher in the dissection group (258 ± 92 min) than in the elective surgery group (197 ± 70 min, p < 0.001), likely because of the longer cooling and rewarming time needed for circulatory arrest in order to perform an open distal anastomosis. Given the emergent and challenging nature of acute dissection repairs, the dissection group had a higher incidence of complications, including neurological events, pneumonia, prolonged ventilation, gastrointestinal complications, acute renal failure, and new dialysis ( Table 4 , p < 0.05). Length of hospital stay was also longer in the dissection group (10.8 ± 15.1 days) than in the elective surgery group (6.4 ± 5.1 days, p < 0.001).

**Table 3.** Operative parameters.

Variable	Type A Dissection Repair (n = 154)	Elective Proximal Aortic Surgery (n = 457)	***P* -value **
Operation year *	2010 (6, 2007–2013)	2010 (6, 2007–2013)	0.641
Redo surgery	10 (6%)	68 (15%)	0.007
Mitral valve surgery	2 (1%)	13 (3%)	0.284
Tricuspid valve surgery	0 (0%)	3 (1%)	0.313
Ascending aortic replacement only	92 (60%)	383 (84%)	< 0.001
Ascending and hemi-arch replacement	51 (33%)	70 (15%)	< 0.001
Ascending and total arch replacement	11 (7%)	4 (1%)	< 0.001
Composite valve graft root replacement	24 (16%)	191 (42%)	< 0.001
Aortic valve replacement	6 (4%)	171 (37%)	< 0.001
Aortic valve repair	120 (78%)	3 (0.7%)	< 0.001
Valve sparing root replacement	1 (1%)	10 (2%)	0.214
Cross-clamp time (min)	104 ± 47	130 ± 50	< 0.001
Cardiopulmonary bypass time (min)	258 ± 92	197 ± 70	< 0.001

* Median (interquartile range). Nominal data are presented as frequency (n) and percentage of the total population and were analyzed using Pearson Chi-square or Fisher’s exact tests. Continuous data are presented as mean ± standard deviation and were analyzed using two-tailed paired Student’s t-tests. Year of operation was analyzed with a median test.

**Table 4.** Postoperative outcomes.

Variable	Type A Dissection Repair (n = 154)	Elective Proximal Aortic Surgery (n = 457)	*P* -value
Neurological events	18 (12%)	4 (1%)	< 0.001
Pneumonia	17 (11%)	9 (2%)	< 0.001
Prolonged ventilation	94 (61%)	58 (13%)	< 0.001
Gastrointestinal events	10 (6%)	8 (2%)	0.003
Acute renal failure	23 (15%)	14 (3%)	< 0.001
New dialysis	10 (6%)	6 (1%)	< 0.001
Surgery to discharge (days)	10.8 ± 15.1	6.4 ± 5.1	< 0.001

Nominal data are presented as frequency (n) and percentage of the total population and were analyzed using Pearson Chi-square or Fisher’s exact tests. Continuous data are presented as mean ± standard deviation and were analyzed using two-tailed paired Student’s t-tests.

### Survival

**Figure 1.**
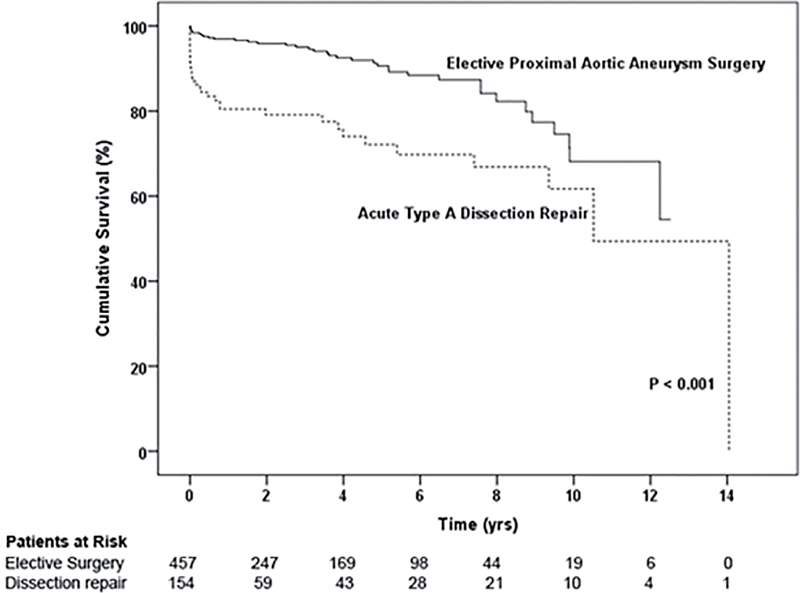
Patient survival for ascending aortic dissection and elective proximal aortic surgery groups.

**Figure 2.**
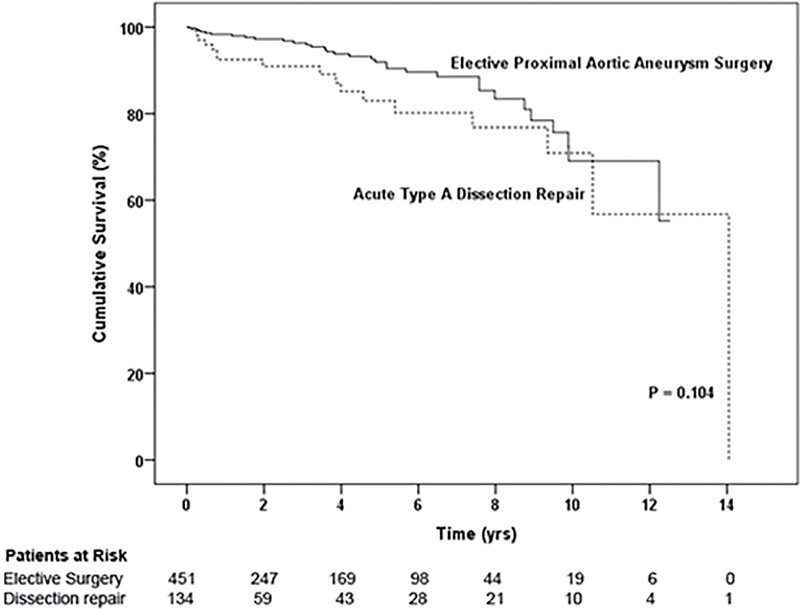
Patient survival for ascending aortic dissection and elective proximal aortic surgery groups after censoring aortic-related deaths in the first year.

Mean follow-up was 2.78 ± 3.61 years for the Type A dissection repair group and 3.32 ± 3.23 years for the elective ascending aortic aneurysm surgery group. The 30-day and 14-year mortality rates were 13% and 24%, respectively, for patients with acute Type A dissection and 1.5% and 8.3%, respectively, for patients who underwent elective ascending aortic aneurysm surgery. Although the dissection group had poorer survival than the elective surgery group ( Figure 1 , p < 0.001), there was no group difference in conditional survival when aortic-related deaths over the first year were censored ( Figure 2 , p = 0.104).

## Discussion

Although coronary angiography was not routinely performed prior to Type A dissection repairs at our institution, we found a very low incidence (2 out of 154 patients, 1.3%) of missed coronary artery disease that became symptomatic soon after surgery and required subsequent coronary intervention. We found another two (1.3%) patients who likely had coronary artery disease in evolution at the time of dissection repair, with lesions not becoming symptomatic until 5 and 7 years postoperatively. Although there likely was atherosclerotic coronary disease that was missed during the acute surgical management of an acute Type A dissection, these lesions seemed to remain mostly silent.

In our Kaplan-Meier survival analysis, there was no difference in long-term conditional survival when early aortic deaths were censored. Given the 1–2% per hour early mortality from acute Type A dissection 8 , the mortality risk of surgical delay for coronary catheterization outweighs the 1.3% risk of missed significant native coronary artery disease requiring early postoperative intervention. Our two patients with early postoperative PCI for missed atherosclerotic coronary artery disease presented with stable angina and shortness of breath on exertion. No patient presented with MI after the initial dissection repair, suggesting that any missed coronary artery disease was likely well collateralized or not severe enough to cause significant hemodynamic issues.

Delays in surgery leading to patient demise in Type A dissection have been well documented by several clinicians. Glower et al. found that 10 out of 91 (11%) patients with DeBakey Type I or II aortic dissections died before an operation could be done 9 . Rizzo et al. found that preoperative coronary angiography led to delays in surgical intervention and increased acute Type A dissection mortality 6 . In an Italian multicenter study where routine preoperative coronary angiography was performed, 23 out of 242 (10%) patients who were considered surgical candidates died before the operation 10 . However, with increasing availability of hybrid operating rooms in the endovascular era, there are greater opportunities in many facilities for intraoperative angiographic coronary evaluation. The negative impact of contrast agents on postoperative renal function needs to be justified. Furthermore, even without routine preoperative coronary angiography, we found a greater baseline incidence of postoperative acute renal failure and new dialysis in the dissection group. Impaired pre- and postoperative renal function can occur from renal malperfusion due to dissection flap obstruction or systemic hemodynamic instability.

However, there may be subpopulations of dissection patients who could benefit from coronary angiography. Indeed, a history of coronary artery disease is associated with increased risk for in-hospital death following Type A dissection repair 11 . Kern et al. found a potential survival benefit of preoperative coronary angiography in patients with Type A dissection with significant clinical history suggestive of coronary artery disease 12 . Interestingly, patients with prior open heart surgery presenting with acute Type A dissection infrequently have tamponade or hemodynamic collapse, which may be due to scar tissue providing support for mediastinal structures. Therefore, coronary angiography may be justified in the preoperative management of these patients, particularly if evaluation of previous CABGs is needed 13 .

Although instrumentation of the dissected aorta for coronary angiography may increase the risk of further aortic complications, we have not experienced any complications from this procedure. Penn et al. found that concomitant CABG at the time of emergent aortic surgery had no effect on in-hospital mortality, and defining coronary anatomy before surgical intervention had no effect on overall CABG rate, likely because 74% of CABGs at the time of surgical repair were for known coronary dissection and not chronic atherosclerotic coronary artery disease 7 . Of the coronary malperfusions in our cohort, four out of five patients died from cardiogenic shock due to myocardial ischemia and MI. This is consistent with a previous finding that a need for concomitant CABG for an evolving myocardial infarct is predictive of postoperative mortality 14 .

The arguments for preoperative coronary angiography include the opportunity to graft a critical stenosis while the patient is on cardiopulmonary bypass during aortic repair to improve the likelihood of successful weaning, to avoid perioperative MI, and to improve survival. However, we had only five (3.2%) cases of perioperative MI, of which four were thought to be due to coronary dissection and malperfusion. Although coronary malperfusion associated with Type A dissection is uncommon, its outcome is often fatal 4 8 15 . No MIs were confirmed to be the result of underlying atherosclerotic coronary artery stenosis. Therefore, routine preoperative coronary angiography to address this disease process is of questionable value in improving postoperative survival.

Creswell et al. reported that the prevalence of atherosclerotic coronary artery stenosis >50% was 34.8% in patients with acute ascending aortic dissection 1 . As ascending aortic aneurysm diseases are precursors to Type A aortic dissection, we believe that electively operated ascending aneurysm patients constituted a reasonable control for the coronary disease in the dissection group. Although routine coronary catheterization was not performed for our dissection patients, we found a 19% concomitant CABG rate in our elective ascending aneurysm surgery population. This percentage is lower than that of Creswell et al., as our criterion for CABG was >70% stenosis for all vessels except for left main disease, where we graft >50% stenosis. Of the 34.8% of patients with coronary artery disease, Creswell et al. found a 75% incidence of single vessel disease and 25% incidence of triple vessel disease in the preoperative angiography group of his acute dissection series 1 . This finding is similar to that in our elective surgery group, in which 82% of CABGs involved two or fewer grafts. Assuming this is representative of the dissection population, the low incidence of multivessel disease may explain the high rate of successful cardiopulmonary bypass weaning in dissection patients despite likely missed underlying significant coronary artery disease. Alternatively, vascular collateralization with chronic coronary compromise may maintain adequate myocardial perfusion and contractility without causing myocardial ischemia or symptoms.

Although aortic dissection patients had a higher early mortality than elective ascending aortic aneurysm surgery patients due to the challenging pathology and emergent nature of the disease, we found no group difference in long-term survival when aortic-related deaths in the first year were censored from the analysis. This suggests that missed coronary artery disease at the time of acute dissection repair does not limit long-term survival. Indeed, the four patients with coronary artery disease in the dissection group who needed subsequent intervention were managed successfully with PCI in a semi-elective manner. Therefore, as angiography is not needed to establish the diagnosis of acute Type A dissection given the advantages of modern tomographic imaging, the benefit of angiography to evaluate coronary artery disease is likely not worth the considerable risks and delay associated with performance of the test, for which no survival benefit can be demonstrated.

The conclusions of this study are limited by its retrospective nature, which has inherent limitations and biases. The lack of routine preoperative coronary angiography in the dissection group precludes accurate assessment of underlying atherosclerotic coronary artery disease. Inferences made for dissection patients by extrapolating the burden of coronary artery disease in the elective ascending aortic aneurysm surgery group may not be valid. Without comprehensive follow-up of the entire patient population, it is possible that patients received subsequent coronary intervention without our knowledge. Causes of intraoperative myocardial ischemia in some cases were unclear and may have been due to coronary button complications, coronary dissection, or underlying atherosclerotic disease. We also do not have data on the clinical presentation or myocardial function of patients with Type A dissection who did not undergo surgery because they were not surgical candidates, refused surgery, or died prior to operation. Patient age and incidence of significant underlying atherosclerotic coronary artery disease may have been higher in this population that did not undergo surgery. Although we found no difference in conditional survival after censoring aortic-related mortalities in the first year, it is possible that our dissection population was too small to detect increased mortality due to complications of residual Type B dissections or missed coronary artery disease at the time of surgery.
